# Defending or Remaining Passive as a Bystander of School Bullying in Sweden: The Role of Moral Disengagement and Antibullying Class Norms

**DOI:** 10.1177/08862605211037427

**Published:** 2021-08-10

**Authors:** Robert Thornberg, Tiziana Pozzoli, Gianluca Gini

**Affiliations:** 1 Linköping University, Linköping, Sweden; 2 University of Padua, Italy

**Keywords:** moral disengagement, antibullying norm, bullying, defending, bystander

## Abstract

The overall aim of the present study was to examine whether moral disengagement and perceptions of antibullying class norms at individual level and at class level were associated with defending and passive bystanding in school bullying among school-age children. More specifically, we investigated the extent to which moral disengagement would contribute to explain defending and passive bystanding, after controlling for sex and perceptions of antibullying class norms at individual level and at class level. A total of 789 Swedish students (aged 10-14) from 40 middle school classes filled out a self-report survey. The findings revealed that girls and students who were less prone to morally disengage, and who perceived that their classmates endorsed more antibullying norms, were more likely to defend victimized peers. Students who were more inclined to morally disengage and perceive that classmates do not condemn bullying were more likely to act as passive bystanders. In addition, classes with higher levels of antibullying class norms were more likely to show higher rates of defending and lower rates of passive bystanding compared to the other classes. The findings suggest that schools and teachers need to develop educational strategies, methods, and efforts designed to make students aware of moral disengagement and to reduce their likelihood of morally disengaging in bullying situations. The present findings also point to the importance of teachers establishing class rules against bullying together with the students.

## Introduction

Bullying refers to repeated aggressive, offensive, or inhumane behavior directed at individuals who are disadvantaged or less powerful in relation to the perpetrator(s) (Hellström et al., 2021). It is a group phenomenon (Salmivalli, 2010), in which peers are most often present as bystanders (Craig et al., 2000). A bystander is commonly defined as any student who witnesses a bullying incident (Polanin et al., 2012; Thornberg et al., 2017b; Yun & Graham, 2018). Bystanders may behave as defenders, meaning that they try to help or support the victim, but they may also respond as outsiders or passive bystanders, and thus remain neutral and keep out of the bullying process. They may even take the bullies’ side as a laughing and reinforcing audience, or by assisting the bullies (Salmivalli, 2010). In the present study, we focus on defending and passive bystanding.

How bystanders act is significant since research has shown that the more often classmates side with the bullies and fail to defend the victims, the higher the prevalence of bullying in the school class, whereas greater defending at class level is linked with less bullying (Kärnä et al., 2010; Nocentini et al., 2013; Salmivalli et al., 2011; Thornberg & Wänström, 2018). Bullying rates vary considerably across countries, and Sweden has been found to have the lowest rates of school bullying in previous cross-national studies (Chester et al., 2015; Craig et al., 2009), but recent national reports indicate a worrying increase in bullying across Swedish schools (Bjereld et al., 2020) and the reason for this is still unknown.

Despite research showing that the majority of school-aged children and adolescents hold antibullying attitudes and think bullying is wrong (Boulton et al., 1999; Thornberg, 2010; Thornberg et al., 2017a), a high number of students do not take action against bullying as bystanders (Craig et al., 2000; Pouwels et al., 2018; Salmivalli & Voeten, 2004; Waasdorp et al., 2018). Whether or not they defend the victim depends on both individual and contextual factors (for a review, see Lambe et al., 2019), and the immediate peer context in which bullying takes place is a significant source (Salmivalli, 2010). The current study aimed to deepen our understanding of how moral disengagement, antibullying class norms, and individual perceptions of antibullying class norms are related to defending and passive bystanding in school bullying among school-aged children.

## Moral Disengagement, Defending, and Passive Bystanding

As outlined in the social cognitive theory of moral agency (Bandura, 2016), *moral disengagement* refers to a set of self-serving cognitive distortions whereby people convince themselves that an immoral action is not immoral and, therefore, not in conflict with their moral standards. In this way, self-regulated mechanisms can be deactivated, and moral self-sanctions can be disengaged. As a result, people can engage in a certain inhumane behavior or refrain from a certain humane and prosocial behavior (such as helping someone in need or distress) without feelings of remorse or guilt. Examples of moral disengagement mechanisms include moral justification (using worthy ends or moral purposes to sanctify immoral behavior), euphemistic labeling (labeling the immoral behavior in a way that makes it sound less negative or more respectable), diffusion of responsibility (diluting personal responsibility due to the presence of other people), dehumanization (stripping the victim of human qualities and equal values), and blaming the victim (believing that the victim deserves his or her suffering).

Children and youth who score higher in terms of moral disengagement more often engage in aggression and bullying (for meta-analyses, see Gini et al., 2014; Killer et al., 2019), and less often defend victims in bullying situations (Gini, 2006; Jiang et al., 2022; Mazzone et al., 2016; Pozzoli et al., 2016; Thornberg et al., 2015, 2017b; for meta-analyses, see Killer et al., 2019; Ma et al., 2019). Research on the link between moral disengagement and passive bystanding is still scarce, and the findings are inconsistent. Some studies have found a negative association (Gini, 2006; Thornberg & Jungert, 2013), while others have identified a positive association (Doramajian & Bukowski, 2015; Gini et al., 2015; Jiang et al., 2022; Sjögren et al., 2021; Thornberg et al., 2017b), whereas Mazzone et al. (2016) found a nonsignificant association, which was also the outcome in Killer et al.’s (2019) meta-analysis. Further research on the association between moral disengagement and passive bystanding is therefore needed. One aim of the current study is to contribute to filling this gap by analyzing the role of moral disengagement in both defending and passive bystanding.

Moral and immoral behavior cannot be reduced to individual psychology, but the social cognitive theory (Bandura, 2016) assumes a *triadic codetermination*, which means that human behavior is produced and regulated by a complex and continual interplay between personal, behavioral, and environmental influences. However, there is limited research that examines whether moral disengagement is still associated with various bystander behaviors when social environment factors, such as group norms, and individual perceptions of social environment factors, such as perceived group norms, are taken into consideration. To overcome this limitation of the current literature, considering the triadic codetermination and the possible tension between the trait-like (habitual personal influence) and situated (environmental influence) aspects of moral disengagement (Bandura, 2016), we examined whether individual students’ levels of moral disengagement contribute to explain defending and passive bystanding in school bullying over and above social norms for or against bullying in the immediate social environment at school (i.e., their school class).

### Antibullying Class Norm

In Sweden, as in many other countries, elementary students usually remain in a single school class with the same classmates for the full school day and for more than one year. This unit or formal group of students is termed *class* in the current study. We focus on students nested into school classes, since we consider this microsystem (c.f., Bronfenbrenner, 1979) to be the most significant source of influence with which students have direct contact in their everyday school life.

All social groups, including school classes, are regulated by *norms*, which can be defined as “consensual standards that describe what behaviors should and should not be performed in a given context” (Forsyth, 2006, p. 12). In their theoretical work on social norms, Cialdini et al. (1990, 1991) suggest a distinction between injunctive norms and descriptive norms, and this social norm typology has been extensively adopted in research on peer groups, interactions, and relationships in childhood and adolescence (Lilleston et al., 2017; Sentse et al., 2015; Veenstra et al., 2018). Injunctive norms refer to what group members approve and disapprove of (what ought to be done), and are usually measured by aggregating individual attitudes within the group of reference. Descriptive norms refer to what group members actually do (what is commonly done), measured by aggregating individual behaviors within the group (Veenstra et al., 2018). Previous research has examined how descriptive class norms and injunctive class norms of bullying are associated with defending (Peets et al., 2015; Pouwels et al., 2019; Pozzoli et al., 2012b; Yun & Graham, 2018).

Within a social group, however, it is also important to consider that what individuals believe other members of their group think about an issue influences their behavior, particularly in relation to bystander situations (e.g., so-called pluralistic ignorance—see Bierhoff, 2002). That is, instead of aggregating individual students’ attitudes toward bullying (i.e., the injunctive norm) at class level, another type of antibullying class norm is the aggregate of students’ perceptions of how widespread antibullying attitudes are among their classmates. Because of the gap between attitudes and behaviors, there is a risk that individual attitudes will remain nonvocalized and hidden from other group members, which, in turn, might weaken their influence on other group members’ behavior. Even though injunctive antibullying class norms have been associated with greater defending (Pozzoli et al., 2012b; Yun & Graham, 2018) and less passive bystanding (Pozzoli et al., 2012b), the effects might be weakened due to invisibility. For example, Sentse et al. (2015) found that individual antibullying attitudes and descriptive antibullying class norms were associated with bullying behavior, but not injunctive antibullying class norms.

Within social cognitive theory, group-level constructs such as collective agency, collective efficacy, and collective moral disengagement are not understood as the mere sum of the individual members’ agency, self-efficacy, or moral disengagement (Bandura, 1997, 2016; Gini et al., 2015; Thornberg et al., 2019). Bandura (2016, p. 13) argues that “a group’s belief is not simply the sum of the individual members’ beliefs. Interactivity produces emergent effects. It is people who make up a group acting coordinately on shared belief.” In the current study, the antibullying class norm is thus not the sum of individual classmates’ antibullying attitudes but the shared belief within the school class of how widespread such attitudes are among the classmates. Like collective efficacy and collective moral disengagement, it is a shared belief that is produced by the group dynamics of the school class. This group-level construct can therefore be understood as a *group’s collective belief about the degree to which an antibullying norm is shared by the group*.

To date, few studies have examined how an antibullying class norm (as a group collective belief about the group) influences bystander behaviors. In their study, Salmivalli and Voeten (2004) asked students to rate their expectations of what their classmates would think and how they would respond if someone in their class were to act as a defender or probully (reinforcer/assistant). The scores were aggregated at class level. This antibullying class norm (expecting negative consequences of probullying and positive consequences of defending) was found to be associated with greater defending in the sixth grade, but not in the fourth and fifth grades.

Pozzoli et al. (2012) constructed a class norm regarding how to behave as a bystander in bullying, by measuring and then aggregating at class level students’ expectations of the degree to which their classmates think they should defend the victim or remain a passive bystander if they witness bullying. Although this antibullying class norm correlated with the prevalence of defending (positively) and passive bystanding (negatively) at class level, it only predicted passive bystanding (negatively) and not defending in the full regression models. However, Kollerová et al. (2018) used a similar construct of antibullying class norm and found that it predicted defending over a period of six months among early adolescents.

Finally, Lucas-Molina et al. (2018) measured antibullying class norms by asking students about their perceptions of their classmates’ antibullying attitudes and then aggregating the scores at class level. As expected, they found that antibullying class norms were associated with greater defending. However, even though it might be plausible to hypothesize that an antibullying class norm—measured as the collective belief of the school class about the degree to which an antibullying norm is shared by the school class—is positively linked with defending, more research is needed due to the lack of studies. Moreover, further research is also needed to understand whether antibullying class norms are useful for explaining differences in the level of passive bystanding behavior in the class.

### Individual Perception of Antibullying Class Norm

Like several other social psychological and social developmental theories, such as social cognition or information processing models (Bosacki, 2016; Crick & Dodge, 1994; Moskowitz, 2005), interactionist theories (Charon, 2001; Hewitt & Shulman, 2011), and decision-making models of bystander behavior (Bierhoff, 2002; Dovidio et al., 2006; Latané & Darley, 1970), social cognitive theory assumes that the way individuals perceive and interpret social situations will affect their behaviors when they interact in these situations (Bandura, 1997, 2016). Thus, not only group norms but also individual perceptions of group norms might be a powerful influence on individual behaviors.

Pozzoli et al. (2012b) argue that children’s perceptions of whether—in the context in which bullying takes place—their peers consider bullying to be desirable or undesirable may affect their own reactions to bullying. Accordingly, previous studies have demonstrated that perceived peer pressure to disapprove of bullying and stand up for the victim is related to greater defending and less passive bystanding (Pozzoli & Gini, 2010; Pozzoli et al., 2012a, 2012b). In addition, Kollerová et al. (2018) found that a similar construct predicted defending over a six-month timespan. Therefore, we hypothesized that students’ individual perceptions of antibullying class norms are positively associated with defending and negatively associated with passive bystanding. Since class norms such as individual-level perceptions and class-level aggregates may have unique effects on bystander behaviors, these two levels were assessed simultaneously (Pozzoli et al., 2012a, 2012b).

### The Present Study

The overall aim of the present study was to examine whether moral disengagement and perceptions of antibullying class norms, at individual level and at class level, were associated with defending and passive bystanding in school bullying among school-age children. More specifically, we investigated the extent to which moral disengagement would contribute to explain defending and passive bystanding, after controlling for perceptions of antibullying class norms at individual level and at class level. Sex was added as a control variable, since previous research has shown that girls are more inclined to defend victims than boys (for a meta-analysis, see Ma et al., 2019). There are, however, inconsistent findings concerning the link between sex and passive bystanding (Doramajian & Bukowski, 2015; Pöyhönen et al., 2012; Pozzoli & Gini, 2013; Thornberg & Jungert, 2013).

Deduced from the reviewed literature, at individual level we hypothesized that being a boy and moral disengagement would be negatively associated with defending, whereas individual perceptions of antibullying class norms would be positively associated with defending. In addition, we hypothesized that individual perceptions of antibullying class norms would be negatively associated with passive bystanding. With reference to the mixed findings in the literature, whether sex and moral disengagement were associated with passive bystanding was examined in an exploratory manner. At class level, we hypothesized that antibullying class norms would explain between-class differences in defending and passive bystanding, meaning that classes with a higher antibullying class norm would show more defending and less passive bystanding. Finally, we tested the potential moderating role of antibullying class norms on the links between individual perceptions of antibullying class norms and the two behaviors (i.e., we tested for cross-level interactions). We expected that this link would be stronger in classes with higher levels of antibullying class norms than in classes with lower levels; that is, we hypothesized that a class normative climate that condemns bullying would magnify the effect of individual perceptions of antibullying norms on defending and passive bystanding.

## Method

### Participants and Procedure

Participants were recruited from 40 middle-school classes at 18 public schools in Sweden. A nonprobability two-step sampling was used in the study. First, a purposive sampling of schools was carried out, which led to the inclusion of the 18 schools (two rural schools, four schools in three small towns, and thirteen schools in different neighborhoods within two medium-sized Swedish cities) representing various sociogeographic and socioeconomic positions. In the next step, we conducted a convenience sampling of students in grades 5-6 at each school. These grades were selected because Swedish national surveys have reported the highest prevalence of bullying in this age group (Swedish National Agency for Education, 2019).

The original sample consisted of 907 students (445 [49%] girls and 462 boys). Parents were informed about the study goals and procedure, and gave their consent for their children to participate in the study (parental consent reached 88%). All the participants were asked for their own consent in addition to parental consent. Two students did not participate because they did not want to, and twelve students were excluded from the study because they did not complete the questionnaire since they were absent due to sickness or for other reasons unknown at the time of data collection.

Thus, the final sample consisted of 789 students (389 girls [49%] and 400 boys; *M*_age_ = 11 years, 9 months; *SD*_age_ = 6 months; age range: 10-14), resulting in a participation rate of 87%. Neither socioeconomic status nor ethnic background data were directly measured in the study; however, the purposeful sampling procedure resulted in schools located in neighborhoods with different socioeconomic statuses, representing lower, middle, and upper-middle classes. Based on information from the schools, the vast majority of the participants have a Swedish ethnic background.

Students completed a questionnaire during regular school hours under the supervision of trained master’s degree students, who explained the study procedure, data confidentiality, and the possibility to withdraw from the study at any time without being penalized, and assisted those participants who needed help (e.g., by providing reading support and clarifying specific items or words in the questionnaire). At the end of the questionnaire administration, the students were thanked for their participation. The participants responded anonymously to the questionnaire.

### Measures

*Defending and passive bystanding behavior.* Students were presented with two situations in order to cover their behavior as bystanders during both physical and verbal bullying (Thornberg et al., 2015). The first question read: “When one or more students repeatedly hit, kick, or harshly shove another student to make them sad, what do you usually do?” The second question was: “When one or more students repeatedly tease another student to make them sad, what do you usually do?” After each question, the students answered four items assessing defending behavior (“I try to make them stop”; “I try to comfort the targeted person”; “I try to defend the targeted person”; “I tell them to stop fighting with/teasing the student”) and four items measuring passive bystanding behavior (“I do nothing special”; “I pretend I don’t see what’s happening”; “I just walk away”; “I don’t watch but carry on doing my own business”) using a 5-point scale (0 = never, 1 = seldom, 2 = sometimes, 3 = usually, 4 = always). Given that passive bystanding items were specifically developed for this study, a second-order confirmatory factor analysis for nested data was performed with four first-order (defending in verbal bullying; defending in physical bullying; passive bystanding in verbal bullying; passive bystanding in physical bullying) and two second-order (defending behavior; passive bystanding behavior) latent variables. The results showed a good fit with the data: χ^2^ (120) = 4293.90, *p* < .001; CFI = .92; TLI = .91; RMSEA = .064 [90%CI = .058-.071]. For each student, we averaged the eight items measuring defending (α = .94; 95% CI = .94-.95) and the eight items measuring passive bystanding (α = .93; 95% CI = .92-.94).

*Moral disengagement in bullying.* In order to measure students’ propensity to morally disengage in bullying situations, an 18-item scale was administered (*Swedish Moral Disengagement in Bullying Scale*; Thornberg & Jungert, 2013; Thornberg et al., 2015). Examples of items were: “People who get teased don’t really get too sad about it,” “Saying mean things to a certain person a couple times a week doesn’t matter. It’s just about joking a little with the person,” and “If you can’t be like everybody else, you have to blame yourself if you get bullied.” Participants rated each item on a 7-point scale, where 1 means “not true at all” and 7 means “very true,” and the mean of the scores was computed (α = .90; 95% CI = .89-.91).

*(Perceived) antibullying class norm.* For the current study, we developed a six-item scale to measure students’ perceptions of how the majority of their classmates judge bullying. Participants read the following introduction: “What do you think most of your class would think if one or more classmates did the following things to another student each week?” This was followed by six items: teasing and calling the person names; beating and kicking the person; excluding the person from the group; spreading bad rumors or lying about the person; shoving the person so hard that it hurts; and making fun of or joking about the person in a way they did not seem to like. Participants evaluated each item using a 4-point scale (1 = “absolutely OK,” 2 = “a bit OK,” 3 = “a bit bad,” 4 = “very bad”). A multilevel confirmatory factor analysis was used to test the monofactorial structure of the scale (χ^2^ [30] = 2781.36, *p* < .001; CFI = .99; TLI = .98; RMSEA = .044; SRMR value for within level = .023; SRMR value for between level = .015). For each participant, scores from the six items were averaged (α = .90; 95% CI = .89-.91) so that higher scores indicated higher perceived antibullying class norms. Moreover, the aggregate score of this scale at class level—that is, the average score of all class members—was computed and provided a measure of antibullying class norm.

### Analytic Techniques

Two models were tested using multilevel path analysis with a Bayesian estimator in Mplus 8.4 (Muthén & Muthén, 2017). Multilevel modeling allowed us (a) to take into account that students were nested within classes; (b) to consider both within- and between-class variability; (c) to test associations between variables at individual and class levels separately; and (d) to examine whether the associations between individual variables varied on the basis of class characteristics.

Specifically, in the first multilevel model, we tested how moral disengagement, perceived antibullying class norms, and sex were associated with defending and passive bystanding at individual level. Moreover, antibullying class norms were examined as being associated with defending and passive bystanding behavior at class level. In the second model, we added the cross-level interaction between perceived antibullying class norms (individual level and class level). All the predictors at individual level were class-mean centered, while the predictor at class level was grand-mean centered.

## Results

Descriptive statistics and correlations among study variables are presented in Table 1 (individual level) and Table 2 (class level). The intraclass correlation coefficients were quite large for both defending (ICC = .235) and passive bystanding (ICC = .293).

**Table 1.** Means, Standard Deviations, and Correlations Among Variables at Individual Level.

	*M*	*SD*	1.	2.	3.
1. Defending	2.67	1.04	–		
2. Passive bystanding	1.12	.97	–.69	–	
3. Moral disengagement	1.85	1.85	–.41	.33	–
4. Perceived antibullying class norms	3.46	.60	.50	–.42	–.34

*Note. N* = 789. All *p*s < .001.

**Table 2.** Means, Standard Deviations, and Correlations Among Variables at Class Level.

	*M*	*SD*	1.	2.
1. Defending	2.71	.55	–	
2. Passive bystanding	1.08	.56	–.91	–
3. Antibullying class norms	3.48	.39	.84	–.87

*Note. N* = 40. All *p*s < .001.

In the first model (Table 3), we tested the role of moral disengagement and perceived antibullying class norms in both defending and passive bystanding behavior at individual level, controlling for sex. Moreover, the model examined the role of antibullying class norms at class level. At individual level, the results showed that sex and perceived antibullying class norms were positively and significantly associated with defending behavior, while moral disengagement showed a negative link with defending and a positive association with passive bystanding. In addition, perceived antibullying class norms were negatively, albeit weakly, associated with passive bystanding. Moreover, an antibullying class norm significantly explained between-class variability in defending (positively) and passive bystanding (negatively). Thus, girls and students who were less prone to morally disengage and who perceived that their classmates endorsed more antibullying norms were more likely to defend victimized peers. By contrast, students who were more inclined to morally disengage and perceive that classmates do not condemn bullying were more likely to act as passive bystanders. Finally, classes with higher levels of antibullying class norms were more likely to show higher rates of defending and less likely to report passive bystanding compared to the other classes. Overall, the first model explained a significant quote of variance (*p* < .001) of the two behaviors at both individual level (defending: *R*^2^ = .25; passive bystanding: *R*^2^ = .10) and class level (defending: *R*^2^ = .45; passive bystanding: *R*^2^ = .72).

**Table 3.** Multilevel Path Analysis Predicting Defending and Passive Bystanding Behavior.

	Model 1						Model 2					
	Defending			Passive Bystanding			Defending			Passive Bystanding		
	Est.	Post. *SD*	95% CI	Est.	Post. *SD*	95% CI	Est.	Post. *SD*	95% CI	Est.	Post. *SD*	95% CI
Individual effects												
Gender (girls)	.22***	.06	.12/.34	–.08	.05	–.19/.03	.22***	.06	.11/.34	–.08	.06	–.20/.02
Moral disengagement	–.32***	.04	–.40/–.25	.26***	.04	.17/.33	–.30***	.04	–.37/–.21	.25***	.04	.17/.33
Perceived antibullying class norms	.44***	.06	.29/.56	–.11^*^	.06	–.22/.02	.46***	.08	.33/.62	–.12^*^	.06	–.23/.02
Class effects												
Antibullying class norms	.59***	.13	.30/.85	–1.01***	.14	–1.32/ –.71	.82***	.13	.53/1.05	–.99***	.14	–1.30/ –.73
Perceived antibullying class norms × antibullying class norms							.44**	.16	.12/.77			

*Note.* Unstandardized coefficients are reported; est. = estimate; post. *SD =* posterior standard deviation; one-tailed *p* value: *p < .05, **p < .01, ***p < .001.

In the second model, the interactions between individual perceptions of antibullying class norms and antibullying norms at class level on defending and passive bystanding were considered. First, we tested whether the relationships of the individual perceptions of antibullying class norms with defending and passive bystanding behavior varied across classes (i.e., we tested for significant random slopes with TYPE = two-level random). The results indicated that this was true for defending (*B* = .46, posterior *SD* = .07, *p* < .001, 95% CI = .33-.62), but not for passive bystanding (*B* = –.11, posterior *SD* = .08, *p* = .10, 95% CI = –.27 to .05). Therefore, we only tested the hypothesis that the relationship between individual perceptions of antibullying class norms and defending behavior was moderated by antibullying class norms. To follow-up the significant interaction (Table 3), we created new parameters to explore the relationship between individual perceptions of antibullying class norms and defending behavior at high (+1 *SD*) and low (–1 *SD*) levels of antibullying class norms (Aiken & West, 1991). The results showed that this relationship was stronger in classes with higher levels of antibullying class norms (*B* = .70, posterior *SD* = .11, *p* < .001, 95% CI = .46-.93) compared with classes with lower levels of antibullying class norms (*B* = .35, posterior *SD* = .10, *p* < .001, 95% CI = .14-.56). The effect of antibullying class norms on the strength of the relationship between perceived antibullying class norms and defending behavior is depicted in Figure 1.

## Discussion

The current study was the first to test whether moral disengagement, individual perceptions of antibullying class norms, and sex at individual level, and antibullying class norms (as a collective belief of how widespread antibullying attitudes were in the class) at class level were uniquely associated with defending and passive bystanding in school bullying.

### Moral Disengagement

Consistent with previous research (Killer et al., 2019; Ma et al., 2019) and our hypothesis, moral disengagement was negatively associated with defending, and this association remained significant even when controlling for sex, perceived antibullying class norms (individual level and class level). A theoretical explanation based on social cognitive theory would be that children who are low in moral disengagement would be more inclined to persist with their moral agency by being influenced by their moral standards and maintaining a more intact and consistent self-regulatory process. Their higher self-awareness, self-monitoring, and tendency to engage in self-approval and self-sanction, depending on how they behave (Bandura, 2016), are not so easily weakened when exposed to real or perceived peer pressure, in terms of perceived antibullying norms shared by classmates. Their habitual pattern of a low level of moral disengagement may therefore still make them prone to defend victims.

In the present findings, moral disengagement was positively associated with passive bystanding. The idea that higher levels of moral disengagement can influence students to remain passive and try to stay outside when witnessing bullying is also in accordance with social cognitive theory (Bandura, 2016), since moral disengagement is assumed to make it easier for people to refrain from helping and protecting someone in distress without experiencing feelings of remorse or guilt. Nevertheless, a few studies have examined the relationship between moral disengagement and passive bystanding, and the findings have been inconsistent (Doramajian & Bukowski, 2015; Gini et al., 2015; Jiang et al., 2022; Mazzone et al., 2016; Sjögren et al., 2021; Thornberg & Jungert, 2013). There might be a number of possible explanations for the inconsistency in the literature. Methodological, cultural, and age differences are possible causes. Other variables that are not included in studies might function as confounders. For example, qualitative studies reveal that students consider many possible motives and reasons for intervening or not intervening, and a common reason for not intervening is the fear of being attacked and even bullied by the bullies and losing social status if they intervene (Forsberg et al., 2018; Strindberg et al., 2021; Thornberg et al., 2018). Such factors might explain why certain students who are low in moral disengagement still refrain from helping and thus remain passive as bystanders. Students who are low in defender self-efficacy are inhibited from defending and more inclined to act as passive bystanders, even though they display a low level of moral disengagement (Thornberg & Jungert, 2013). The inconsistent findings can thus be discussed in relation to Oberman’s (2011) distinction between unconcerned passive bystanders (high in moral disengagement) and guilty passive bystanders (low in moral disengagement). Nevertheless, it should be noted that, in our study, moral disengagement contributed to explain passive bystanding over and above individual perceptions of antibullying class norms and the class’ collective belief about the degree to which an antibullying norm is shared by the class.

### Antibullying Class Norms

The current findings showed that both antibullying class norms and individually perceived antibullying class norms were uniquely associated with defending and passive bystanding, after controlling for sex and moral disengagement. Thus, our study suggests that students are more inclined to defend a victim of bullying and less likely to remain passive if they belong to a school class that is characterized by a strong antibullying class norm, but also if students perceive that their class is characterized by a strong antibullying class norm. Nevertheless, the negative association between individual perceptions of antibullying class norms and passive bystanding should be viewed with great caution. Indeed, although the one-tailed *p* value was significant, the Bayesian CI suggested that this result was absolutely negligible. Finally, we also found that the positive association between perceived antibullying class norms and defending was stronger in classes with higher levels of antibullying class norms.

In accordance with social cognitive theory (Bandura, 2016), our findings suggest that students’ bystander behavior in school bullying is due to an interplay between personal and contextual influences. All social groups are regulated by group norms (Brown, 2000; Forsyth, 2006), and how individuals perceive and interpret their group and various social situations in this group setting will influence and guide their behavior (Bierhoff, 2002; Bosacki, 2016; Crick & Dodge, 1994; Hewitt & Shulman, 2011; Moskowitz, 2005). The present findings suggest that both normative peer pressure and student-perceived normative peer pressure contribute to explaining their defender behavior, while the former in particular—but not the latter—seems to influence their passive bystanding behavior. Our study thus contributes to the literature on how class norms of bullying are linked with bystander behavior (Peets et al., 2015; Pouwels et al., 2019; Pozzoli et al., 2012a, 2012b; Yun & Graham, 2018) by showing the importance of antibullying class norms conceptualized as classes’ collective beliefs about the degree to which antibullying norms are shared by the class, in addition to previous research on descriptive and injunctive class norms of bullying. Our findings further confirm the positive relationship between antibullying class norms and defending found in Lucas-Molina et al.’s (2018) study, but go beyond their findings by also demonstrating the negative relationship between antibullying class norms and passive bystanding. Hence, the current findings support the growing evidence that bullying is a group phenomenon (Hymel et al., 2015; Salmivalli, 2010), the importance of the class microsystem (Doll et al., 2004; Saarento et al., 2015) and its class norms (e.g., Peets et al., 2015; Pouwels et al., 2019; Pozzoli et al., 2012a, 2012b), and the interplay between individual and class-level contextual factors to explain various bystander behaviors in bullying (Gini et al., 2015; Thornberg et al., 2017b).

### Limitations and Practical Implications

Some of the limitations of the present study are worth mentioning. A major limitation is the cross-sectional nature of our data. This means that we cannot draw causal conclusions or pinpoint the direction of the identified associations among the variables. For example, it is not clear whether moral disengagement is a predictor of defending, or whether defending predicts moral disengagement. It is also possible that the relationships identified in the study are bidirectional or reciprocal, which social cognitive theory actually assumes by referring to triadic codetermination (Bandura, 1997, 2016). For instance, and in accordance with social cognitive theory, students who initially remain passive when witnessing bullying may gradually disengage self-sanctions for such behavior, which allows them to maintain their passive bystanding with fewer and fewer feelings of guilt. At the same time, these students may continue to disengage from self-sanctions for passive bystanding, which in turn allows them to increase their passive bystanding. Therefore, future studies should adopt a longitudinal design to examine possible bidirectional, longitudinal associations across moral disengagement, antibullying class norms, individual perceptions of antibullying class norms, and various bystander behaviors in bullying. Another limitation is that we have used self-reported data, which are vulnerable to social desirability bias, memory distortion, and intentionally exaggerated responses. Self-reported data might also inflate variable associations due to shared method variance.

Finally, a note of caution needs to be sounded regarding the generalization of the findings. The sample of children from certain areas of Sweden may or may not be similar to the population of children and adolescents whom readers primarily work with or are interested in studying. We have applied a purposeful sampling of schools to include various sociogeographic and socioeconomic backgrounds, and then a convenience sampling of students in grades 5-6 at each school. In other words, we have not conducted a randomized sampling from the Swedish population of students in grades 5-6, which further limits the generalizability of this age group population even in Sweden. Although the current study examined the role of sex, it did not examine diversity variables such as ethnicity, socioeconomic status, religion, age, ability, language, and culture. In addition, other measures of behaviors associated with moral disengagement and defending such as antisocial behavior, delinquency, self-control, and self-efficacy were not included or controlled for in the current study. Thus, future studies should test the interrelations among the variables found in our study in other cultural contexts (both in Sweden and in other countries) and with various subgroups of students. Further research could also investigate whether ethnicity, socioeconomic status, nationality, ability, and other diversity variables moderate the effects. Cronbach (1975, p. 125) argues that social phenomena are too variable and context-dependent to permit universal and confident empirical generalizations. “When we give proper weight to local conditions, any generalization is a working hypothesis not a conclusion.” Thus, our findings (like social research findings in general) should be considered as partial, provisional, and fallible working hypotheses that need to be further examined and tested through future studies in various contexts and populations.

These limitations aside, the present study has some practical implications. In line with the growing body of research (Gini, 2006; Jiang et al., 2022; Mazzone et al., 2016; Pozzoli et al., 2016; Thornberg et al., 2015, 2017b), our findings suggest that schools and teachers need to develop educational strategies, methods, and efforts designed to make students aware of moral disengagement and to reduce their likelihood of morally disengaging in bullying situations. For example, Wang and Goldberg (2017) evaluated a classroom-wide bullying intervention in which children’s storybooks about bullying were used. Each mechanism of moral disengagement was included throughout the intervention. Classroom conversations were held around these mechanisms. The educational aims included making students aware of moral disengagement and making them understand that bullying cannot be justified under any circumstances. The findings from this evaluation showed that this intervention was promising, as both moral disengagement and victimization decreased in the treatment classrooms.

Teachers can teach adolescent students about—and draw their attention to—the presence of moral disengagement mechanisms in historical and contemporary cases of crimes against humanity (e.g., the Nazi Holocaust and other incidents of genocide, terrorism, torture, and hate crimes toward and discrimination against minorities). They can then encourage students to compare these cases with their own everyday life to increase their ability to be aware of whether the very same mechanisms operate (albeit less conspicuously) in their own school, classroom peer groups, and other familiar social settings, and what effects they might have on themselves and others (cf. Brabeck et al., 1994). This could be a way to teach students to recognize, uncover, and resist moral disengagement in their everyday life in order to increase their moral agency, sense of personal responsibility, and compassions for others.

Furthermore, the present study points to the importance of teachers establishing class rules against bullying together with the students, in accordance with, for instance, the Olweus Bullying Prevention Program (Olweus & Limber, 2007). Our findings suggest that, if teachers are successful, the prevalence of bullying will probably be lower in the class because classmates would be more inclined to help, defend, and support a victim if they happened to witnessing bullying. As previous research has revealed, greater defending at class level is linked with less bullying (Kärnä et al., 2010; Nocentini et al., 2013; Salmivalli et al., 2011). Bandura (2016, p. 39) argues that the responsibility for counteracting bullying “cannot be placed solely on the shoulders of a few children who are morally heroic bystanders,” but “requires systematic changes in the school culture involving key constituencies at all levels of influence.” One element of such an antibullying school culture is to establish strong antibullying norms in each class.

## Author Biographies

**Robert Thornberg**, PhD, is a professor of education in the Department of Behavioural Sciences and Learning at Linköping University in Sweden. His major areas of research interest are school bullying and bystander behaviors of bullying and peer victimization, particularly in relation to moral and social processes, among children and adolescents.

**Tiziana Pozzoli** is PhD in developmental psychology. She is currently a research assistant in the Department of Developmental and Social Psychology, University of Padova, Italy. Her research interests concern individual and contextual variables associated with different behavior during bullying episodes, focusing especially on bystanders’ behavior.

**Gianluca Gini**, PhD, is a professor of developmental psychology at the Department of Developmental and Social Psychology, University of Padua, Italy. His research interests include the group dynamics underlying bullying, moral cognition and bullying, and consequences of peer victimization.
